# Does women’s caste make a significant contribution to adolescent pregnancy in Nepal? A study of Dalit and non-Dalit adolescents and young adults in Rupandehi district

**DOI:** 10.1186/s12905-018-0513-4

**Published:** 2018-01-22

**Authors:** Hridaya Raj Devkota, Andrew Clarke, Shanti Shrish, Dharma Nanda Bhatta

**Affiliations:** 1 Kidasha, Pokhara, Nepal; 2 Kidasha, London, UK; 3 Lancashire Care NHS Foundation Trust, Lancaster, UK; 4 Tribhuvan University, People’s Dental College, Kirtipur, Nepal; 5 Department of Epidemiology and Public Health, University College London (UCL), 1 – 19 Torrington Place London WC1E 6BT, London, UK

**Keywords:** Adolescent, Pregnancy, Caste, Nepal

## Abstract

**Background:**

Adolescent pregnancy is a public health concern worldwide. There are disparities in the occurrence of adolescence pregnancy in different social groups and settings; however, few studies have focused on the contribution of a woman’s caste in early pregnancy in Nepal. This study aimed to examine the association between caste and adolescent pregnancy; and investigate factors that influence this among women of Dalit and non-Dalit caste groups.

**Methods:**

A cross sectional survey among 457 women, age between 14 and 24 years was carried out in Rupandehi district of Nepal. Bivariate and multivariate logistic regression analysis using a stepwise entry method was performed to assess the association of women’s caste, ethnicity and other socio-demographic and individual factors with early pregnancy.

**Results:**

Over two thirds of the participants (69%) were pregnant during adolescence. The highest percentage of adolescent pregnancies were reported among women from J*anajati* groups (77%) and the lowest in B*rahmin/Chhetri* caste groups (45%); while 72.5% of women from D*alit* caste groups reported adolescent pregnancy. When adjusted for demographic and individual variables, early pregnancy was less likely among women who were from B*rahman/Chhetri* (OR: 0.60; 95% CI: 0.30, 1.22) and M*adhesi/Muslim* (OR 0.56; 95% CI: 0.23, 1.36) compared to women from the D*alit* caste, but multivariate regression analysis found none of these were statistically significant. Women who had secondary level education (OR: 0.34; 95% CI: 0.17, 0.65), had married after 17 years of age (OR: 0.02; 95% CI: 0.01, 0.14) and had attended fairs/clubs (OR: 0.40; CI: 0.21, 0.79) were significantly less likely to experience early age pregnancy. Women who drank alcohol (OR: 5.18; 95% CI: 1.02, 26.32) were significantly more likely to become pregnant during adolescence compared to women who did not drink alcohol.

**Conclusions:**

Women’s caste had no direct contributory role in the early pregnancy of the sample. Education, age at marriage and individual behaviours were the key contributing factors. Reducing the number of adolescent pregnancies requires addressing the factors that lead to and perpetuate child marriage; keeping girls within education systems for longer; increase the knowledge and control of girls over their own reproductive health and planning; and actions that promote gender respect within relationships, decision-making and negotiation among both girls and boys.

**Electronic supplementary material:**

The online version of this article (10.1186/s12905-018-0513-4) contains supplementary material, which is available to authorized users.

## Background

Adolescent pregnancy (defined as pregnancy <20 years of age) and childbirth is a social and public health concern with a wide range of physical, psychological, social and economic consequences for the adolescent girls, their babies, families and communities [1, 2]. Adolescent pregnancies have increased risks of delivery complications for the mother and for stillbirth, pre-term births, low birth weight and high mortality [3]. It is a major contributor to child and maternal mortality in low and middle-income countries, where 95% of adolescent pregnancies occur [1, 2].

Around 16 million girls age between 15 and 19 years and one million under 15 years give birth every year, accounting for 11% of all births worldwide but 23% of the global burden of disease from pregnancy and childbirth. The highest rate of adolescence pregnancy is reported in Central African Republic at 229/1000 and the lowest in South Korea at 2.9/1000 [1, 2].

In Nepal, adolescents (10–19 years’ group) comprise of 24% of the total population. Over the fifteen years from 1996 to 2011 the adolescent pregnancy rate decreased from 24% to 17% [4] however, the median age at first pregnancy remained 16.2 years [5].

Contributing factors for adolescent pregnancy include early marriage, early sexual debut, socio-cultural and economic factors, the educational status of the girl and her parents, sexual violence and peer pressure [5–9]. The practice of early marriage and expectations to have a child soon after is a social norm in South Asian cultures [10, 11]; and reaching menarche regarded as a signal to begin childbearing [12]. The legal minimum age of marriage for woman in Nepal is 18; however, child marriage is still very common and 52% of women get married before they turn 18 [13] with 19% of 20–24 year old women giving birth to their first child before their 18th birthday [14].

Whilst many risk factors for adolescent pregnancy in Nepal and other developing countries occur within marriage [2] this does not apply to all groups. Only 1% of woman aged 15–24 years reported pre-marital sex in the Nepal Demographic and Health Survey (2011) but among adolescent girl factory workers 16% of unmarried girls had been sexually active [15].

Peer pressure, alcoholism and illicit drug use are important contributors to teenage pregnancy in both developing and developed countries [10, 16] and this is a factor in the Nepali context too [17], although adolescents experiencing abuse and coerced sex are at higher risk [18, 19]. But the attitude of young Nepali’s towards sexual relationships has also changed with exposure to electronic media, the internet and social networks all impacting on the rise of pre-marital sex [8].

In Nepal, social inequalities, a family’s economic status, gender roles, parental and girl education levels are important predictors of adolescent pregnancy, and family characteristics have a strong influence on a young person’s sexual behaviour and likelihood of young pregnancy [20]. The prevalence of adolescent pregnancy is significantly higher in women from lower social classes compared to those from higher classes [21, 22]. Similarly, religion and ethnicity can be a marker for differing sexual practices, use of family planning and use of abortion. Hindu girls in Nepal also have children at an earlier age than Buddhist girls [9].

Adolescence and puberty are periods of great physiological and emotional change and development [23, 24]. Erikson de-constructed this, identifying eight stages of psychological development that occur during adolescence, each of which present potential crises or turning points, including that of ‘identity’ verses ‘identity confusion’; including the internalization of ethnicity, sexual and gender orientation, and socio-economic factors. Erikson proposed that successful transition through this enables movement towards healthier adult lives, whereas ongoing identity confusion leads to prolonged ‘identity searching’ and a state where dropping out of school and moodiness are more likely. Young people who struggle with issues relating to their emerging identity are more likely to engage in choices and lifestyles associated with increased sexual behaviours and resulting pregnancy, but can be effectively encouraged to postpone sexual activity if they have strong support systems [24, 25].

“The developmental assets framework” [26] identifies twenty internal and twenty external factors believed to be healthy building blocks that help young people to grow up healthy, caring and responsible. Internal assets are those that enhance a commitment to learning, positive values such as integrity and responsibility, social competencies and positive identity such as self-esteem. External assets include those that build support from the youth’s family and community, such as the care provided by family, empowerment, and availability of role models.

Young people from all ethnicities and backgrounds that have greater risk-taking behaviours such as alcohol and substance use and exposure to or involvement in violence, are also associated with greater risk-taking behaviours relating to sex [27, 28]. The degree to which an individual has an internal locus of control strongly influences their behaviour and amount of effort and persistence they may employ to achieve their chosen options [29]. Those with a strong sense of internal control tend to more successful in achieving their choices, which further reinforces their sense of empowerment; and vice versa. This also translates into their relationships and sexual health; adolescents who encounter strong external control and feelings of powerlessness are less likely to consistently use birth control methods [30–32].

Previous studies have explored adolescent pregnancy in developing countries including some that focused on ethnic minorities [33], however a review of the literature reveals a lack of evidence relating to associations between caste and adolescent pregnancy, and none that specifically focus on adolescent pregnancy amongst girls of historically disadvantaged caste groups in Nepal such as Dalit.

Population composition and description is complex in Nepal and sub-group definition, in both official and usual-use, is less consistently delineated than in many countries, resulting in sub-groups based on caste, ethnicity and religion being counted and cited in parallel. This study aimed to examine the association between caste and adolescent pregnancy; and to investigate other factors influencing early pregnancy among Dalit and non-Dalit women in a southern district of Nepal. Following “The developmental assets framework”, socio-demographic, economic and family related variables were considered external factors; and variables related to individual behaviours were considered internal factors.

## Methods

### Study design and setting

A cross sectional survey was carried out during February 2016 among Dalit and non-Dalit caste women in a district of southern Nepal. The district has a population of 880,196 people of which 50.89% are female. Women aged 14–24 years comprise 24% of the female population in the study district with an annual pregnancy rate of 39% [34]. Out of 125 recorded ethnic and indigenous groups in Nepal, the study district population comprises upwards of 95 different groups and indigenous inhabitants including 28 sub-groups of Dalits. The majority of people (78%) live in rural villages, though the urban population is growing fast. In terms of caste breakdown by the 2011 census, the Rupandehi population comprised of 12.03% Dalits, 24.45% Janajatis, both regarded as socially and economically disadvantaged castes, and 15.21% Brahmins and 5.62% Chhetries, caste groups historically more advantaged and powerful [34].

Healthcare services in the district are delivered through five Primary Health Care Centres (PHCC), six Urban Health Clinics (UHC), sixty-four Sub/Health Posts (S/HP) and 2 hospitals. In addition to the government health sector, there is a wide network of NGO and private sector services providing private hospitals, nursing homes, clinics and pharmacies/drug shops [35].

### Sample and sampling procedure

Women attending maternal child health clinics who met the inclusion criteria of being 14–24 years of age, currently pregnant or given birth in the last 12 months were recruited for interview. Nineteen trained female enumerators interviewed 457 Dalit and non-Dalit women privately in the health facilities using a pre-tested structured questionnaire.

A two stage sampling method was used to select health facilities. In the first stage, the district was stratified into seven clusters based on electoral constituencies and in the second stage, nine health facilities, primary health care centres, health posts and sub-health posts were selected randomly, including two hospitals.

### Tool/instrument

A structured questionnaire was designed on the basis of previous adolescent pregnancy studies as well as by adapting validated questions from the Nepal Demographic and Health Survey 2011 [14]. The questionnaire (Additional files 1 and 2) included questions about socio-demographic factors in the first section, family related information in the second section and questions about respondent’s individual behaviour in the final section. The tool was developed in the Nepali language (Additional file 2), field-tested and then adjusted before implementation.

### Variables and description of measures

Table 1 shows study variables, variables coding and description of variables. Previous studies on adolescent pregnancy and related theories guided the selection of variables. The external variables were demographic and family related, including women’s caste and ethnicity, age, place of residence, education, occupation, age at marriage, type of marriage, husband’s education, husband’s occupation and household wealth. Intrinsic variables relating to the women’s individual behaviour were age at first sex and indicators of personal freedoms such as frequency of attendance at local fairs or clubs, going movies or cinema and frequency of alcohol consumption.

**Table 1:** Descriptions of variables

Variable	Coding	Description
Socio-Demographic		
Caste/Ethnicity	1 = Dalit	Woman’s reported caste and ethnic group
	2 = Brahmin/Chhetri	
	3 = Jana Jaati	
	4 = Madhesi/Muslim	
Age of women	Continuous	Completed age of the woman at the time of survey in years
Place of residence	1 = Urban	Woman’s place of residence at the time of survey
	2 = Rural	
Respondent’s education	0 = Illiterate	Number of years of education completed by the woman
	1 = Primary (up to 5 grade)	
	2 = Secondary and above (6 +)	
Respondent’s occupation	1 = Unemployed	Occupation of woman at the time of survey
	2 = Farmer/Laborer	
	3 = Employed/Business	
Age at marriage	1 = Under 16 years	Age of woman at marriage in years
	2 = 17 years. and above	
Type of marriage	1 = Love/Self 2 = Arranged	Self-reported; A love marriage is chosen partner herself and self-get married
Family Background		
Husband’s education	0 = Primary or less/Illiterate	Number of years of education completed by the women’s husband
	1 = Secondary and above (6 +)	
Husband’s occupation	1 = Unemployed/Labor/Farmer	Occupation of women’s husband at the time of survey
	2 = Employed/Business	
Household wealth index	1 = Poor 2 = Middle 3 = Rich	Whether or not the family have: - Live stocks: Buffalo, Cow/Bull, Horse/Mule, Goat/Sheep, Chicken/Duck/Pigeon, Pigs - Household items: Electricity, Motorbike, Rikshaw, Bull cart, Bicycle, Phone, TV, Radio, Fan, Computer, Refrigerator - Own house - Type of roof - Toilets and its type - Source of water use
Individual Behaviour		
Age at first sex	1 = 11–16 years 2 = 17 years and above	Completed age of woman at the time of first sexual intercourse
Often/sometime attends fair/Club	0 = No 1 = Yes	Woman attending local fairs and clubs
Often/sometime goes to movie	0 = No 1 = Yes	Often goes to movie
Often/sometime drinks alcohol	0 = No 1 = Yes	Alcohol consumption by the woman

The caste and ethnicity variables reflect the socio-cultural structure of Nepalese society in which Dalits are at the bottom of the caste hierarchy and considered poor, marginalized and with few opportunities and limited social mobility. Brahmins are at the top of the hierarchy followed by Chhetries and are considered as privileged groups with better access to socio-economic and life opportunities [36].

A wealth index was constructed of 24 indicators of possession of household assets and characteristics (listed below). Except for roofing and source of water indicators, all other variables were dichotomized assigning 0 or 1 with a total score of 24. In the case of roofing materials, the household was assigned the weight 1 with natural/non-durable roofing, 2 with durable roofing (Tin/Jasta/Tile) and 3 for those with concrete roofing. Similarly, household using surface water or shallow well were given the score 1, those using tube well were assigned 2 and who were using pipe water were given 3 score. The sample was then divided into quintiles from one as poorest to five the richest. However, the lowest and highest two groups were collapsed to make three categories of wealth ranking in order to have enough cases in each category for analysis.

### Analysis

The field data was entered using Epi-Data 3.1 to minimize entry error and then transferred to SPSS (Version 22.0) for analysis, following cleaning for missing data and consistency. Four hundred fifty-four cases were included in the analysis with three cases excluded due to incomplete information.

The dependent variable in multivariate analysis was the first pregnancy of women below 20 years of age, coded as binary variable. We conducted bivariate analysis using logistic regression to obtain unadjusted odds ratios to examine the effect of a broad range of variables on first pregnancies below the age of 20. Then multivariate logistic regression analysis was conducted using a stepwise entry method to find the independent effects of variables on pregnancy. We first entered the interest variable (caste) in the model 1 and then added socio demographic and family related variables in model 2. In model 3, we entered all variables making a full model to observe the possible effects. Prior to including variables in the regression models, we conducted a collinearity diagnostic test on all explanatory variables to check collinearity among the variables. None had a variance inflation factor (VIF) more than 4.201.

## Results

### Characteristics of participants

Table 2 shows out of 454 participants, over half were Dalits. A quarter (24%) were Jana Jaati, one-sixth (16%) were Brahmin/Chhetri and about one-tenth (10%) were Madhesi and Muslim. The vast majority of the participants (82%) lived in rural areas. Approximately one in ten women reported themselves as illiterate, while one-third stated they had completed education up to primary level and the majority (58%) stated their education as being secondary level and above. Over one-third of women had married below the age of sixteen; just under two-thirds of their marriages had been arranged and more than one-third (38%) had chosen their partner themselves (love marriages). Just over half (58%) reported that their husbands were employed or engaged in business. Two-thirds of women reported their husband’s education level secondary and above. About one-third participants had their sexual debut at 16 years or younger, 77% reported that they often attend local fair or clubs and 57% often go to movies or the cinema. A small percent (6%) stated that they sometimes consume alcohol.

**Table 2:** Individual background characteristics

Factors	n	%
Caste/Ethnicity	454	100
Dalit	229	50.4
Brahmin/Chhetri	71	15.6
Jana Jaati	109	24.0
Madhesi/Muslim	45	9.9
Place of Residence	454	100
Rural	372	81.9
Urban	82	18.1
Education	454	100
Primary (Up to 5)	135	29.7
Secondary & above (6 - University)	265	58.4
Illiterate	54	11.9
Occupation	454	100
Unemployed	391	86.1
Others (Farmer/Laborer/Business)	63	13.9
Age @ Marriage	451	100
09–16 years	155	34.4
17 years and above	296	65.6
Family Background		
Family Type	454	100
Nuclear	84	18.5
Joint/Extended	370	81.5
Husband’s Education	451	100
Primary or less/Illiterate	146	32.4
Secondary & above (6 - University)	305	67.6
Husband’s Occupation	451	100
Farmer/Laborer/Unemployed	189	41.9
Employed/Business	262	58.1
Wealth Index (Family)	454	100
Lowest wealth groups (<40%)	101	22.2
Middle wealth groups (41–60%)	246	54.2
Highest wealth groups (>60%)	107	23.6
Individual Behaviour		
Type of Marriage	452	100
Love/Self married	170	37.6
Arrange	282	62.4
Age @ First sex	454	100
11–16 years	138	30.4
17 years and above	316	69.6
Attending Fairs/Clubs	454	100
No	106	23.3
Yes	348	76.7
Goes to movie	454	100
No	197	43.4
Yes	257	56.6
Drinks Alcohol	454	100
No	427	94.1
Yes	27	5.9

Table 3 shows that 69% of women had their first pregnancy under the age of twenty. A number of socio-demographic and behavioral factors were observed to be associated with adolescent pregnancy (*P* < 0.05). Among the caste and ethnic groups, Jana Jaati reported the highest (77%) proportion of adolescent pregnancy (OR 1.28, 95% CI 0.75–2.17), followed by Dalits (73%) and Madhesi/Muslim women (69%) (OR 0.84, 95% CI 0.42–1.68). This proportion for Brahmin/Chhetri was the lowest (45%) (OR 0.31, 95% CI 0.18–0.54). Women’s education and age at marriage was found to be statistically significant for early pregnancy (*P* < 0.001). Just under 84% of women with primary education reported they had a pregnancy before they turned 20 years of age. This proportion was less among the women with no education (74%) (OR 0.56, 95% CI 0.26–1.19) and secondary and above education (60%) (OR 0.30, 95% CI 0.18–0.50). Almost all women (97%) who were married at 16 years or earlier had a pregnancy below the age 20, while only 54% women getting married at the age 17 or later had their pregnancy under 20 (OR 0.03, 95% CI 0.01–0.09). The family factors, husband’s education (*P* < 0.01), husband’s occupation (*P* < 0.01) and wealth status (*P* < 0.05) were also found to be significantly associated. Women whose husbands had secondary level education or above (OR 0.53, 95% CI 0.34–0.84) and whose husbands were employed (OR 0.49, 95% CI 0.32–0.75) were also less likely to be pregnant in their teens. 56% of women from the highest wealth group had a pregnancy before the age of twenty, whereas 72% of women in the lowest and middle wealth groups were pregnant as teenagers (OR 1.75, 95% CI 0.98–3.14 and OR 1.69, 95% CI 1.05–2.72 respectively).

**Table 3:** Bivariate analysis result showing association between socio-demographic, individual behavior factors and pregnancy under the age 20

Factors	Pregnancy < 20 years		OR	95% CI	*P* - Value
	n	%			
Caste/Ethnicity	313	68.9			
Dalit (Ref)	166	72.5			
Brahmin/Chhetri	32	45.1	0.311	0.18–0.54	0.000
Jana Jaati	84	77.1	1.275	0.75–2.17	0.371
Madhesi/Muslim	31	68.9	0.840	0.42–1. 68	0.624
Place of Residence	313	68.9			
Urban	58	70.7	1.109	0.66–1.87	0.699
Rural (Ref)	225	68.5			
Education	313	68.9			
Primary (Ref)	113	83.7			
Secondary	160	60.4	0.297	0.18–0.50	0.000
Illiterate	40	74.1	0.556	0.26–1.19	0.131
Occupation	313	68.9			
Unemployed (Ref)	267	68.3			
Others (Farmer/Labor/Business)	46	73	1.257	0.69–2.28	0.452
Age @ Marriage	310	68.7			
09–16 years (Ref)	151	97.4			
17 years and above	159	53.9	0.031	0.01–0.09	0.000
Family Type	313	68.9			
Nuclear (Ref)	61	72.6			
Joint/Extended	252	68.1	0.805	0.48–1.36	0.420
Husband’s Education	310	68.7			
Primary or less/Illiterate (Ref)	113	77.4			
Secondary+	197	64.6	0.533	0.34–0.84	0.006
Husband’s Occupation	310	68.7			
Farmer/Labor/Unemployed (Ref)	146	77.2			
Employed/Business	164	62.6	0.493	0.32–0.75	0.001
Wealth Index (Family)	313	68.9			
Lowest wealth group	73	72.3	1.752	0.98–3.14	0.059
Middle wealth group	176	71.5	1.689	1.05–2.72	0.031
Highest wealth group (Ref)	64	55.8			
Type of Marriage	311	68.8			
Love/Self	120	70.6	1.143	0.76–1.73	0.525
Arrange (Ref)	191	67.7			
Age @ First sex	313	68.9			
11–16 years	132	95.7	0.061	0.03–0.14	0.000
17 years and above (Ref)	181	57.3			
Attending Fairs/Clubs	313	68.9			
No (Ref)					
Yes	227	65.2	0.436	0.26–0.74	0.002
Goes to movie	257				
No (Ref)					
Yes	171	66.5	0.770	0.51–1.16	0.206
Drinks Alcohol	27				
No (Ref)					
Yes	25	92.6	6.033	1.41–25.83	0.015

Statistically significant individual behaviour factors associated with early pregnancy were the age at first sex (*P* < 0.001), attending fairs and clubs (*P* < 0.01) and alcohol consumption (*P* < 0.05). Women who used to attend local fairs and clubs were less likely to get pregnant in their teens than those not attending (OR 0.44, 95% CI 0.26–0.74). The study also showed that women who sometimes consumed alcohol were more likely to be pregnant in their teens (OR 6.03, 95% CI 1.41–25.83).

Table 4 shows the multivariate analysis result with adjusted odds ratio. We fitted all the factors into three models to control the confounding effect of the variables. The interest variable of the study, caste and ethnicity, was entered into the first model. In model two, other demographic and family related variables were added along with caste, and the final model consisted as a full model entering all variables together.

**Table 4:** Multivariate analysis results with adjusted Odds Ratio

Factors	Adjusted OR (95% CI)					
	Model 1		Model 2		Model 3	
	OR	95% CI	OR	95% CI	OR	95% CI
Caste/Ethnicity						
Dalit (Ref)						
Brahmin/Chhetri	0.311***	0.18–0.54	0.564	0.28–1.12	0.603	0.30–1.22
Jana Jaati	1.275	0.75–2.17	1.748	0.94–3.26	1.643	0.87–3.10
Madhesi/Muslim	0.840	0.42–1.68	0.669	0.29–1.56	0.555	0.23–1.36
Place of Residence						
Urban			0.774	0.40–1.49	0.839	0.43–1.62
Rural (Ref)						
Education						
Primary (Ref)						
Secondary			0.328**	0.17–0.63	0.335**	0.17–0.65
Illiterate			0.429	0.18–1.05	0.374*	0.15–0.95
Occupation						
Unemployed (Ref)						
Others (Farmer/Labor/Business)			1.159	0.55–2.45	1.298	0.59–2.84
Age @ Marriage						
09–16 years (Ref)						
17 years and above			0.032***	0.01–0.09	0.019***	0.01–0.14
Family Type						
Nuclear (Ref)						
Joint/Extended			0.967	0.50–1.89	0.950	0.48–1.89
Husband’s Education						
Primary or less/Illiterate (Ref)						
Secondary+			1.262	0.66–2.41	1.302	0.67–2.52
Husband’s Occupation						
Farmer/Labor/Unemployed (Ref)						
Employed/Business			0.636	0.38–1.07	0.654	0.38–1. 11
Wealth Index (Family)						
Lowest wealth group			0.890	0.40–1.98	0.761	0.33–1.75
Middle wealth group			1.053	0.58–1.91	1.071	0.58–1.97
Highest wealth group (Ref)						
Type of Marriage						
Love/Self			0.811	0.48–1.37	0.868	0.50–1.50
Arrange (Ref)						
Age @ First sex						
11–16 years					0.578	0.09–3.74
17 years and above (Ref)						
Attending Fairs/Clubs						
No (Ref)						
Yes					0.402**	0.21–0.79
Goes to movie						
No (Ref)						
Yes					1.063	0.61–1.85
Drinks Alcohol						
No (Ref)						
Yes					5.175*	1.02–26.32
Intercept		2.635***(0.969)		107.667***(4.679)		324.140***(5.781)
Model Chi-square		22.202***		143.849***		155.884***
Degree of freedom		3		14		18
-2Log likelihood		540.357		415.718		403.684
Nagelkerke R-square (%)		6.7%		38.4%		41.1%
Percent correctly classified		70.5%		73.6%		76.0%

Wald Chi square *P < 0.05, **P < 0.01, ***P < 0.001

After the control of confounders, only four factors, women’s education, age at marriage, attending local fair and clubs and alcohol consumption were retained in the final model as having statistically significant associations (*P* < 0.05) with the women’s pregnancy under the age 20 (Table 4).

## Discussion

The study hypothesized that women’s caste correlated with early pregnancies; and that Dalit women, being socio-economically disadvantaged and more marginalized, would have a higher proportion of pregnancies at an early age. Initial bivariate analysis appeared to reinforce this, however multivariate analysis found no significant association and the study did not support the hypothesis, revealing that women’s caste and ethnicity have no relation to early pregnancy. This contradicts some of the previous studies conducted in Nepal and other countries [33, 37] but also highlights the importance of accounting for multiple factors when using data to generate new contextual understanding for decision-making and policy.

The study found women’s education level, age at marriage, lack of personal freedoms such as attending fairs and clubs, and alcohol consumption, were significantly associated with early pregnancy. None of the family factors included in the study showed a significant association. Women with primary education were more likely to become pregnant as an adolescent than either those with secondary or higher education, or women with no education at all. The finding for women with secondary level education conforms to a large body of evidence that retaining girls in education develops self-esteem, empowerment and motivation to delay pregnancy [21, 37, 38]. Women with secondary and higher level education may also be better informed about the risks and consequences of early pregnancy as well as protective mechanisms to avoid it. However, the finding for women with no education was less expected. It could be hypothesized that girls who are not enabled to go to school at all may have more restrictions on their lives than girls able to attend primary school, and that this may have an impact on their family and social contacts.

Early sexual debut and early marriage are regarded as important contributing factors for adolescent pregnancy [8, 11]. This study contradicted that early engagement in sexual activity contributed to early pregnancy. The findings of this study about the contribution of early marriage to early pregnancy was consistent with those previous studies, with the same social and cultural pressures found for early child-bearing soon after marriage [11, 37, 39].

Two behavioural factors were found to be associated with early pregnancy, attending local fairs and clubs and consumption of alcohol, although interestingly, the association was reverse between pregnancy and having personal freedoms to attend fairs and clubs. Those attending fairs and clubs were less likely to experience early pregnancy and it may be that this group have greater control, autonomy, and freedom in their decision-making. They may also have better opportunity to access family planning and other protective measures than their counterparts. Dalit women have been found to enjoy more autonomy and freedom and are more empowered within their families and relationships than non-Dalit women [40, 41].

As found in previous studies, women consuming alcohol were found more likely to experience early pregnancy than non-consuming [42], although the percentage reporting alcohol consumption in this study was small. It is difficult to separate individual factors from their social and family context. Issues of self-esteem, locus of control, education and economic status are often reported as inter-related with disadvantage and family functioning and have been linked to teenage pregnancy [11, 12, 16, 42]. However, the findings of this study indicate that intrinsic factors about the individual also have an important bearing on early pregnancy. Adolescent’s feeling towards sexuality and sexual behaviours, specifically in the formation of sexual partnership, sexual negotiation, ability in decision making in relation to the risk of pregnancy and feeling of responsibility within the partnership are poorly understood. Further studies are needed to explore those issues in the Nepali context and the complex relationship between social, cultural, economic and internal factors, and adolescence pregnancy.

## Limitation

The study had some limitations. Firstly, the sampling of participants was limited to those attending the health facilities and the results may not reflect the characteristics of non-attendants or be generalized to those. Secondly, the cross-sectional nature of the study limits the ability to establish a causal direction between independent and dependent variables. Moreover, the study did not include information to help understand the complexity of socio-cultural aspects, which may influence adolescent’s sexual behaviour and childbearing. Thirdly, there could be under reporting about sex, sexual behaviour and pregnancies by survey participants due to it being a culturally sensitive issue and with stigma associated with engaging in sexual activity at an early age. However, even with these limitations, the study results are helpful in understanding adolescent pregnancy and the factors associated in the context of fast and profound behavioural change in this population group in Nepal. Future studies should address these limitations and produce a more rigorous and in-depth understanding of adolescent pregnancy.

## Conclusion

The study identified that women’s caste had no direct contributory role in early pregnancy. However, individual behaviour, education level and early marriage were key contributing factors. Effective implementation of existing laws and policies to deter child marriage and retain girls in school would help to reduce early pregnancy. In addition, comprehensive interventions should be implemented that address not only socio-economic aspects but also cultural expectations traditional practices that may be harmful. Promotion of sex and relationships education in schools, together with interventions that encourage empowerment and gender respect for both girls and boys may make an important contribution towards developing their self-esteem and decision-making ability and delay pregnancy.

## Additional files

Additional file 1: Survey Questionnaire (English). (PDF 552 kb)

Additional file 2: Survey Questionnaire (Nepali Version). (PDF 753 kb)
