# Supplementary material for: Does women’s caste make a significant contribution to adolescent pregnancy in Nepal? A study of Dalit and non-Dalit adolescents and young adults in Rupandehi district
Source: BMC Womens Health. 2018 Jan 22;18:23. doi: 10.1186/s12905-018-0513-4 (PMC5778648; doi:10.1186/s12905-018-0513-4)
Supplement: Supplementary file 1 — Survey Questionnaire (English). (PDF 552 kb) [file 12905_2018_513_MOESM1_ESM.pdf]

QUESTIONNAIRE FOR WOMEN UNDER 24 YEARS HAVING CHILDREN OR CURRENTLY PREGNANT

A	SOCIO-DEMOGRAPHIC INFORMATION	CODE	SKIP PATTERN
1	Name of the respondent	....	
2	Name of VDC/municipality	....	
3	Ward number		
4	Village/Tole	....	
5	Age of respondent	Completed year	
6	What is your caste and ethnicity?	Dalit (Terai) ………………………….….1<br>Dalit (Hill) ………………………….….2<br>Bramhin/Chhetri (Terai) …….…..……3<br>Bramhin/Chhetri (Hill) ….……….….…4<br>Janjati (Terai) ………………….....…….5<br>Janjati (Hill) …………….…………6<br>Other (Specify) ________ 96	
7	What is your religion?	Hindu ……….…………..... ….….…1<br>Buddhist………......................... ...... 2<br>Islam (Muslim) ………..…… …..…..3<br>Isai (Christian).......................... ......…4<br>Other (Specify) ________ 96	
8	Can you read and write?	Yes.………………….……….……....1<br>No …… …………………..…………2	No → GT 11
9	What is the highest grade you completed?	NFE………………………….....……..1<br>Primary (1-5)...….……………….…...….. 2<br>Secondary (6-10/SLC)…..……….......…3<br>Higher secondary (+2)……….............…4<br>Higher education/university ……….......5	4, 5 } GT 12

10	What was the reason to terminate your education?<br>(Ask to those who terminate school before SLC)	Poor household economy……………..1<br>Needed to take care of siblings………….2<br>Failed/unsuccess in exam……………….3<br>Overage than other students……… ……4<br>Marriage…………………………… ……5<br>Sexual harassment……………….……6<br>Other (specify)………………………….. 96	
11	What was your age when you terminated your education?	Completed year [ ][ ]	
12	What is your occupation?	Student………………………………...1<br>Housewife……………..…..…………..2<br>Farmer …...3<br>Daily Wage Laborer………….............4<br>Casual Wage Laborer…......................5<br>Small Trade/Business….......................6<br>Service/Employment…........................7<br>Other (Specify) ________________ 96	
13	What is your current marital status?	Unmarried……...…………….……….1<br>Married ……….............................……2<br>Widowed …….……..………….…3<br>Other (Specify) ________________ 96	
**B**	**FAMILY BACKGROUND**		
14	What is your family type?	Nuclear ……………….………......……1<br>Joint (In-laws)….……….........………...2<br>Extended (aunts/uncles) ……………….3	
15	Who is the household head in your family?	Respondent self ...……………........….1<br>Husband …………………......……….2	

		Father-in-law ………………......……….3<br><br>Mother-in-law ………….....…......…..…..4<br><br>Other (Specify) ________________ 96	
16	What is the highest education level of your husband?	Non Formal Education ……..........….......1<br><br>Primary (1-5)...…………………..........…. .2<br><br>Secondary (6-10/SLC)…..……...........…3<br><br>Higher secondary (+2)……….............4<br><br>Higher education/university …..............5	
17	What is the occupation of your husband?	Unemployed……………………………1<br><br>Farmer ....2<br><br>Wage Labourer..3<br><br>Small Trade/Business................................4<br><br>Service/Employment.................................5<br><br>Self-employed.……………..…………...6<br><br>Other (Specify) ________________96	
18	What is the <u>main</u> income source of your family?	Farming……………………….................1<br><br>Wage labour...2<br><br>Small Trade/Business...............................3<br><br>Service/Employment................................4<br><br>House Rent...5<br><br>Other (Specify) ________________ 96	
19	Do your family own the house?	Yes ...……………….................................1<br><br>No…..……………..................................2	
20	What is the roofing material of your house?	Grass/straw ………………….....……..1<br><br>Tin/Jasta ..……………………....……..2<br><br>Tile/khapada …………………....……..3<br><br>Cement and Concrete...………......……4	

		Plastic/tents ……………………....……5<br>Other (Specify) ____________ 96	
21	Do you have your own toilet at your house or in your compound?	Yes...1<br>No..2	
22	If yes, type of toilet	Temporary...1<br>Permanent..2<br>Other (Specify) __________ 96	
23	Where do you get water?	Piped water (Public)...............................1<br>Piped water (Pvt.)....................................2<br>Tube well (Public)...................................3<br>Tube well (Pvt)...4<br>Well (Public)…………………………..5<br>Well (Pvt.)……………………………..6<br>Other (Specify) ______________ 96	
24	Do your family keep animals?	Yes ……………………....…..………1<br>No ……………....…………....………2	
25	Do your family keep following livestock?<br><br>***(Multiple response)***	Buffalo………......……....…… ☐<br>Cow/bulls ….....................…… ☐<br>Horses/Mule……….....……… ☐<br>Goats/Sheep's…….……...... ☐<br>Chicken/Ducks…..…....…… ☐<br>Pigs........………......………… ☐<br>Other (Specify) __________ 96	
26	Do your household have <u>following household items</u>?	Electricity …….…………...….………1<br>Motorbike ………………....………….2<br>Riksa ……………………....………….3<br>Bull/buff cart ……………….....………4	

	(Multiple response)	Bicycle ……….…………..………..5<br>Mobile phone …………….....….……..6<br>TV ………………………......………..7<br>Radio …………………......………....8<br>Fan ………………….…….....………9<br>Computer..……………......……….10<br>Refrigerator …………………......…….11	
**C**	**PARENT'S BACKGROUND**		
27	What is your parent's caste and ethnicity?	Dalit (Terai) ……………….……….….1<br>Dalit (Hill) ……………………….……2<br>Bramhin/Chhetri (Terai) ……..…..……3<br>Bramhin/Chhetri (Hill) ….……….…..…4<br>Janjati (Terai) …………………...…….5<br>Janjati (Hill) …………….………….6<br>Other (Specify) ______________ 96	
28	Highest Education Level of Your Father	Illiterate …………………......….……1<br>Literate (NFE) ………........…...............2<br>Primary (1-5)...…..…………….........… 3<br>Secondary (6-10/SLC)…..……..........…4<br>Higher secondary (+2)……….............5<br>Higher education/university …..............6	
29	Highest Education Level of Your Mother	Illiterate …………………......….……1<br>Literate (NFE) ………........…...............2<br>Primary (1-5)...…..…………….........… 3<br>Secondary (6-10/SLC)…..……..........…4<br>Higher secondary (+2)……….............5	

		Higher education/university …..............6	
30	What is the main income source of your parents?	Farming ……………………….............1<br>Wage labour........................... ....2<br>Small Trade/Business..............................3<br>Service/Employment...................... ....4<br>House Rent...5<br>Other (Specify) ________ 96	
31	Do your parent's own the house?	Yes ...……………..................................1<br>No…..…………….................................2	
32	What is the roofing material of your parent's house?	Grass/straw ………………….....…….1<br>Tin/Jasta ..…………………….....….…2<br>Tile/khapada …………………......……3<br>Cement and Concrete...……….... ...… 4<br>Plastic/tents ……………………......….5<br>Other (Specify) ________ 96	
33	Do your parent's have own toilet at their house or in their compound?	Yes..1<br>No...2	
34	If yes, type of toilet	Temporary..1<br>Permanent..2<br>Other (Specify) ________ 96	
35	Where do they get water?	Piped water (Public)................................1<br>Piped water (Pvt.).....................................2<br>Tube well (Public)......... ....3<br>Tube well (Pvt)................................ ......4<br>Well (Public)………………………… .5<br>Well (Pvt.)……………………………..6<br>Other (Specify) ________ 96	

36	Did your parent keep animals?	Yes ……………………….....………….1<br><br>No ……………......…………....……….2	
37	Did your parents keep following livestock?<br><br>***(Multiple response)***	Buffalo………......……......…… ☐<br><br>Cow/bulls …......................…… ☐<br><br>Horses/Mule………......……… ☐<br><br>Goats/Sheep's….…….…....... ☐<br><br>Chicken/Ducks…..…......…… ☐<br><br>Pigs........………......………… ☐<br><br>Other (Specify) ________ 96	
38	Do your parent's have following household items?<br><br>***(Multiple response)***	Electricity ………………...….………….1<br><br>Motorbike ………………....………….2<br><br>Riksa ……………………....………….3<br><br>Bull/buff cart ………….…....……….4<br><br>Bicycle …………………….....………..5<br><br>Mobile phone ………………....….….6<br><br>TV ………………………....……….7<br><br>Radio …………………......………....8<br><br>Fan ……………………….....……….9<br><br>Computer..………………......………10<br><br>Refrigerator …………………....……11	
**D.**	**INDIVIDUAL BEHAVIOUR**		
39	Do you ever go to clubs, local fairs, parties, dancing and enjoy?	Yes...1<br><br>No..2 →	GT 41
40	If yes, how often do you go?	Regularly ....1<br><br>Sometimes..2<br><br>Occasionally ....3<br><br>Only once 4	

41	Do you ever go to movie/films?	Yes..1<br>No...2 →	GT 43
42	If yes, how often do you go?	Regularly ....1<br>Sometimes...2<br>Occasionally ....3<br>Only once 4	
43	Do you sometimes drink alcohol?	No/Never..1<br>Only once ....2<br>Occasionally/rarely.......................................3<br>Sometimes..4<br>Regularly 5	
44	Do you smoke?	No/Never..1<br>Only once ....2<br>Occasionally/rarely.......................................3<br>Sometimes..4<br>Regularly 5	
**E**	**MARRIAGE AND PREGNANCY**		
45	What type of marriage you have had?	Love/Self………………..……………....1<br>Arrange………………..……………..2 →	GT 48
46	If love marriage, how did you meet/get your partner	School friend……………..………..……..1<br>Met him through face book/social network………………………....……2<br>Met him through friends………...………3<br>Met him in the clubs…..……………………4<br>Other (Specify)…………………………96	
47	If love marriage, how did you get influence for marriage?	No one (myself)……………………..… .1<br>TV/Film…………………………………2<br>Peer pressure…………………… …….3	

		Mutual understanding…… ……………4<br>Other (Specify) ______________ 96	
48	Who decided for your marriage?	Self ……………………………………1<br>Parents ……………………………….2<br>Other family members ………………3<br>Other (Specify) ______________ 96	→ GT 50
49	Were you asked for your marriage?	Yes.………………………………………1<br>No …… ……………………………………2	
50	What was your age at marriage?<br>(Completed year)	Year [ ][ ]	
51	Did you know the legal marriage age for female in Nepal? Can you tell?	No ……………………………………….1<br>15 Years ……………………………….2<br>16 Years…………………………………3<br>17 Years…………………………………4<br>18 Years…………………………………5<br>19 Years…………………………………6<br>20 Years…………………………………7	
52	Reasons for marriage?<br>**(Multiple response)**	Weak economic condition ……………..1<br>Expensive at late stage ………………..2<br>Culture / costume ……………………...3<br>Illegal relationship/rape/abuse…………4<br>School dropout/failed …………………..5<br>Other (Specify) ______________ 96	
53	Do you know the consequences of early marriage?	Yes.…………………………………….1<br>No ……………………………………..2	→ GT 55
54	What are these consequences? Can you tell?<br>**(Multiple response)**	Drop out of education ………………1<br>Having children earlier………………2<br>Health/Physical effect……………….....3	

		Mental effect……………… …..…....4<br>Lack of independence……… …..….5<br>Other (Specify) ____________ 96	
55	How many children do you have?	Not given birth yet ……………………1<br>One…………………………………….2<br>Two…………………………………….3<br>Three………………………… ……..4	
56	How many times did you have pregnancy?	Total no. of pregnancies ☐	
57	How old were you at your first pregnancy?	Completed years ☐☐	
58	How many pregnancies have you had?	1<br>2<br>3	
59	Which one is this your pregnancy?	1<br>2<br>3	
60	Is it planned/ intended pregnancy?	Yes (as planned)...……….……………1<br>No (against my will).…………………2	
61	In your opinion, what is the main reason why teenagers get pregnant?<br>***(Multiple response)***	Mistake………………………….………1<br>Carelessness…………………………..…2<br>To keep boy friend/girl friend………..…3<br>Benefits………………………………….4<br>Want to become mother…….…………..5<br>Other (Specify) …………………….……96	
62	Who/What influenced you for getting pregnancy?<br>***(Multiple response)***	Peer/friends……………………………1<br>Husband………………………………..2<br>Family pressure..……………………….3<br>Cultural belief…………………………4<br>By medias and social networks ………5<br>Other (Specify) ____________ 96	

63	Did you face any health problem or complication during your pregnancy and giving birth?	Yes……………………………………….1<br>No……………………………………..2	→ GT 64
64	If yes, what problem did you face?	Abortion……………………………..……1<br>Pre-term baby……………………………2<br>Low birth weight baby…………….……..3<br>Complication during delivery……………4<br>Prolapsed uterus…………………………5<br>Vaginal Tear/Injury……….…………..6<br>Disability with baby……………………...7<br>Disability with mother (Fistula)…………8<br>Other (Specify) ________ 96	
65	Did you feel disappointed /sad during pregnancy or after having baby?	Yes………………………………………..1<br>No……………………………………...2	→ GT 66
66	If yes, What did you feel? Probe and write in points	……………………………………………<br>……………………………………………	
67	Did you face any problem in school, family, neighbours, and society because of your early pregnancy?	Yes.…………………….…………….………1<br>No …… …………………...…..…………2	→ GT 68
68	If yes, what problem did you face?	Terminated school/lost education……….1<br>Not accepted by family/parents…………2<br>Shame/humiliation……………………….3<br>Self-isolated………………………………4<br>Economic burden………………………..5<br>Other (specify)…………………………96	
**F**	**KNOWLEDGE ABOUT STD, HIV/AIDS**		
69	Do you know about STDs and HIV/AIDS?	Yes.…………………….…………….………1<br>No …… …………………...…..…………2	→ GT 71

70	How can a person get STDs and HIV?<br><br>**(Multiple response)**	Sexual intercourse ………….........................1<br>Touching to infected.......................................2<br>Sharing go towel/materials used by infected....3<br>Sharing needles/unclean<br>medical equipment……………………..4<br>Blood Transfusion……………………..5<br>During pregnancy……………………..6<br>Mother to child during birth…………..7<br>Mosquito or other insect bites…………8<br>Through breast milk…………………...9<br>Other (Specify) ________ 96	
71	How it can be prevented or avoided getting STDs and/or HIV/AIDS?<br><br>**(Multiple response)**	Not to have sex before marriage ……….1<br>Not to have sex with multiple partners…2<br>Avoid unsafe sex……………………….3<br>Avoid sex completely/abstinence….,…..4<br>Stay faithful to husband………………..5<br>Encourage husband to stay faithful…….6<br>Avoid contaminated blood………..,…..,7<br>Use condoms for every act of<br>sexual intercourse…………………….8<br>Avoid sharing needles………………..9<br>Avoid casual sex…………………,,…10<br>Other (Specify) ________ 96	
**G**	**SEXUALITY, CONTRACEPTIVES & ABORTION**		
72	How old were you when you had first sexual intercourse?	Completed years [ ][ ]	
73	Do you know about contraceptive?	Yes……………..………..……..………….1<br>No …………………..…..……..….…..…..2	→ GT 77

74	Did you use any contraceptive device during your first and subsequence sexual intercourse?	Used in first only………....………………1<br>Used in subsequent intercourse..………….2<br>Used all times before planning pregnancy...3<br>Not used in first time.....................................4<br>Never used..5<br>Other (Specify)...96	
75	If not why you didn't use?	I /my partner did not like to use…....…1<br>It was not available……………...…….2<br>It makes sterility ………………...……3<br>Effect on health & menstruation cycle ………..........................……………4<br>Negative effect on health ……………5<br>It was costly to buy……………..……6<br>Not having money to buy……………7<br>We/I like to have another child…...…8<br>Other (Specify) …………..……….96	
76	Have you ever used emergency contraceptives after intercourse or unprotected sex (econ to prevent conception)?	Yes……………………………………1<br>No……………………………………..2	
77	When did you use it?	6 month before ………………………1<br>Before 7 – 12 month ………………....2<br>Before 13 months …………………….3	
78	Did you have ever abortion?	Yes……………………………………1<br>No……………………………………..2 →	GT 81
79	Why did you do it?	Spontaneous...1<br>Illegal pregnancy...2<br>Early pregnancy & didn't like..................3<br>Husband's pressure....................................4	

		Family pressure...5<br>Health reason ....6<br>Other (Specify) 96	
80	If yes, where did you perform it?	Hospital/Health facility……………......1<br>Private clinic/Pharmacy…………...…2<br>Traditional healers/herbalists……….....3<br>Other (Specify)……………………......96	
81	Do you know the consequences of abortion?	Yes……………………………………1<br>No………………………..……..…2	
82	Do you know the time/weeks within you can do abortion?	Before 12 weeks (Medical abortion).....1<br>Before 18 weeks (Rape case)…………2<br>Anytime (health-risks)………………..3<br>Do not know…………………………..4<br>Other (Specify)……………………….96	
**H**	**WOMEN EMPOWERMENT**		
83	Do you go out to health facility, market or other places from your home alone? Is there any restriction for you to go out?	Yes…………………………......……..1<br>No……………………………....……2	
84	Can you decide yourself to go HF when you get sick or for your pregnancy check up?	Yes…………………………......……..1<br>No……………………………......…..2	→ GT 85
85	If no, who makes the decision?	Husband...1<br>Mother in-laws...2<br>Father in-laws...3<br>Other (Specify)..96	
86	Can you spend money on your own for day to day purchase without permission of other family members/husband or mother-in-law?	Yes…………...……..…………………..1<br>No………………...……………………2	

I	VIOLENCE/ABUSE		
87	Do you feel safe when you are with your family?	Yes……………………………………1<br>No…………………………………….2	GT 88
88	If no, why?	Physical abuse………………………..1<br>Mental abuse/torture…………………2<br>Sexual harassment……………………3	
89	Have you ever been a victim of sexual abuse?	Yes…………………………….……..1<br>No…………………………………....2	GT 93
90	How often do you experience sexual abuse?	Very often……………………………..1<br>Often……………….…………………2<br>Sometimes……………………………3<br>Rarely…………………………………4	
91	Who perpetrated sexual abuse against you?<br>**(Multiple response)**	Employer………………………………1<br>Husband……………………………. …2<br>Other family member…………………3<br>Police…………………………………4<br>Friends…………………………………5<br>Someone from community/neighbours…6<br>Other (Specify) ________ 96	
92	Did you tell this to a responsible adult the last time you were exploited or abused?	Yes ……………………………………..1<br>No……………………………………2	
93	If no, why not?	Nothing gets done…………………......1<br>No one listens……………………..……2<br>Fear of trouble…………………………3<br>Ashamed………………………………4<br>No one to tell…………………………5<br>Other (Specify) ________ 96<br>Do not know…………………….……..99	

J	DISABILITY RELATED		
94	Do you have any disability?	Yes ………………………...….……….1<br><br>No ……………....…………....………2 →	End Interview
95	If yes, what type of disability do you have?	Visual..……………….…….………….1<br><br>Hearing ….…..………………….…......2<br><br>Mental……….…………………….…..….3<br><br>Physical ………..…………….……..…….4<br><br>Multiple……………………………..… 5	
96	When did you get your disability?	By birth ………………….…………….888<br><br>After birth at the age of ………… ☐	

Thank you!
