# Supplementary material for: Does women’s caste make a significant contribution to adolescent pregnancy in Nepal? A study of Dalit and non-Dalit adolescents and young adults in Rupandehi district
Source: BMC Womens Health. 2018 Jan 22;18:23. doi: 10.1186/s12905-018-0513-4 (PMC5778648; doi:10.1186/s12905-018-0513-4)
Supplement: Supplementary file 2 — Survey Questionnaire (Nepali Version). (PDF 753 kb) [file 12905_2018_513_MOESM2_ESM.pdf]

२४ बर्ष भन्दा मुनिका गर्भवति वा बच्चा भएका महिलाहरुलाई सोधिने प्रश्नावलि

प्र.नं	प्रश्न	कोड	स्कीप
**क.सामाजिक, आर्थिक तथा जनसांख्यिक विवरण सम्बन्धि प्रश्नहरु:**			
१	उत्तरदाताको नाम	.... ☐☐☐	
२	नगरपालिका/गा.वि.स नाम	.... ☐☐	
३	तपाईंको घर कति नं वडामा पर्छ ?	वडा नं. .... ☐☐	
४	गाउँ/टोल	....	
५	तपाई कति वर्ष पुरा हुनु भयो ?	उमेर वर्षमा लेख्नुहोस् ☐☐	
६	उत्तरदाताको जात थर ?	दलित (तराई)..१<br>दलित (पहाड)..२<br>ब्राम्हण/क्षेत्री (तराई).................................३<br>ब्राम्हण/क्षेत्री (पहाड)................................४<br>जनजाती (तराई)..५<br>जनजाती (पहाड)..६<br>अन्य (खुलाउनुहोस) ____________ ९६	
७	तपाई कुन धर्म मान्नु हुन्छ ?	हिन्दू ....१<br>बौद्ध...२<br>ईस्लाम (मुस्लिम)....................................३<br>ईसाइ (क्रिस्चियन)..................................४<br>अन्य (खुलाउनुहोस्).......................... ९६	
८	तपाई लेख पढ गर्न सक्नु हुन्छ ?	सक्छु...१<br>सक्दिन...२	
९	तपाईको पढाइको स्तर कति सम्म रहेको छ ?	अनौपचारिक शिक्षा.................................१<br>प्राथमिक (१ देखि ५)...............................२<br>माध्यामिक (६ देखि १०/SLC)...................३<br>उच्च माध्यामिक (+२)............................४<br>उच्च शिक्षा/बिश्व बिद्यालय.....................५	प्रश्न १२ मा जाने

१०	के कारण ले पढाइ छोड्नु भयो ?<br><br>(SLC भन्दा अगाडि बिधालय छोडने लाई सोध्ने)	घरको आर्थिक अवस्था कम्जोर भएर.............१<br>भाईबहिनीको स्याहार सुसार गर्नु परेको.ले.......२<br>परिक्षामा फेल भएकोले..............................३<br>अरु बिद्यार्थीहरु भन्दा मध्यम भएकोले............४<br>बिबाहको कारणले. ....५<br>यौन दुरब्यवहारको कारणले ....६<br>अन्य (खुलाउनुहोस) ९६	
११	पढाइ छोड्दा तपाइको उमेर कति थियो ?	पुरा बर्षमा ☐☐	
१२	तपाईको ब्यवसाय के हो ?	बिधार्थी..१<br>गृहिणी..२<br>किसान..३<br>नियमित ज्याला मजदुरी.............................४<br>कहिले काँही ज्याला मजदुरी.........................५<br>साना ब्यापार ब्यवसाय...............................६<br>जागिर..७<br>अन्य (खुलाउने).......................................९६	
१३	हाल तपाईको श्रीमान कंहाँ हुनुहुन्छ ?	अबिवाहित...१<br>बिवाहित..२<br>बिदुवा..३<br>पारपाचुके/छोडपत्र....................................४	
**ख. पारिवारीक बिवरण प्रश्नहरु**			
१४	तंपाईको परिवारको प्रकार<br>**(संयुक्त- सासु/ससूरा सहित एउटै घरमा बसेको)**<br>**(बृहत-सासु/ससूरा, काका/काकी सहित एउटै घरमा बसेको)**	एकल..१<br>संयुक्त ....२<br>बृहत...३	
१५	तंपाईको परिवारको घरमुली को हो ?	उत्तरदाता आफै ....१<br>श्रीमान...२<br>ससूरा...३<br>सासु...४<br>अन्य खुलाउनुहोस ९६	

१६	तपाईको श्रीमानले कति पढ्नु भएको छ ?	अनौपचारिक शिक्षा...............................१<br>प्राथमिक (१ देखि ५)..............................२<br>माध्यमिक (६ देखि १०/SLC)..................३<br>उच्च माध्यामिक (+२)...........................४<br>उच्च शिक्षा/बिश्व बिद्यालय....................५	
१७	तपाईको श्रीमानको <u>मुख्य पेशा</u> के हो ?	वेरोजगार...१<br>कृषि ....२<br>ज्यालामजदुरी...३<br>सानो व्यापार..४<br>जागिर...५<br>स्वरोजगार..६<br>अन्य (खुलाउनुहोस) ९६	
१८	तपाईको परिवारको <u>मुख्य आम्दानीको</u> श्रोत के हो ?	कृषि.... ....१<br>ज्यालामजदुरी...२<br>सानो व्यापार/व्यवसाय................................३<br>जागिर..४<br>घरभाडा...५<br>अन्य (खुलाउनुहोस) ९६	
१९	तपाईको परिवारको आफ्नो घर छ ?	छ...१<br>छैन...२ →	प्रश्न २१ मा जाने
२०	तपाईको घरको छाना के को छ ?	खर/छवाली...१<br>टिन/जस्ता..२<br>टाईल/खपटा..३<br>सिमेन्ट...४<br>पलास्टिक/टेन्ट..५<br>अन्य (खुलाउनुहोस) ९६	
२१	तपाईको घरमा चर्पी छ ?	छ...१<br>छैन...२ →	प्रश्न २३ मा जाने

२२	यदि छ भने कुन प्रकारको छ ?	अस्थाई...१<br>स्थाई..२<br>अन्य (खूलाउनुहोस) ९६	
२३	तपाईले कहाँको पानी प्रयोग गर्नुहुन्छ ?	पाइपको पानी (सार्वजनिक)...........................१<br>पाइपको पानी (निजी)..................................२<br>टयूव वेल (सार्वजनिक).................................३<br>टयूव वेल (निजी)...४<br>ईनार/कुवा (सार्वजनिक)................................५<br>ईनार/कुवा (निजी).......................................६<br>अन्य (खुलाउनुहोस) ९६	
२४	के तपाईको परिवारले गाईवस्तु पाल्नु भएको छ ?	छ..१<br>छैन...२	
२५	के तपाईको परिवारले निम्न उल्लेखीत जनावरहरु पाल्नु भएको छ ?<br><br>**(वहूउत्तर)**	भैसी.. ☐<br>गाई/गोरु.................................... ☐<br>घोंडा/गधा.................................. ☐<br>भेंडा/बाखा.................................. ☐<br>कुखुरा/हाँस................................. ☐<br>परेवा... ☐<br>सूंगूर .... ☐<br>अन्य (खूलाउनुहोस) ९६	
२६	तपाईको घरमा तलका सामानहरु छन् ?<br><br>**(वहूउत्तर)**	विजुली...१<br>मोटरबाईक..२<br>रिक्सा...३<br>राँगागाडा वा वयलगाडा............................४<br>साईकल...५<br>मोबाइल फोन..६<br>टि.भी..७<br>रेडियो..८<br>पड्खा..९<br>कम्प्यूटर...१०<br>फ्रिज..११	

ग. माइती (अमावावुको) बिवरण सम्बन्धि प्रश्नहरु			
२७	उत्तरदाताको माईत को जात/थर के हो ?	दलित (तराई)..१<br>दलित (पहाड)..२<br>ब्राम्हण/क्षेत्री (तराई)................................३<br>ब्राम्हण/क्षेत्री (पहाड)...............................४<br>जनजाती (तराई).....................................५<br>जनजाती (पहाड).....................................६<br>अन्य (खुलाउनुहोस) ________ .९६	
२८	तपाईंको बुवाले कति पढ्नु भएको छ ?	पद्नु भएको छैन......................................१<br>अनौपचारिक शिक्षा...................................२<br>प्राथमिक (१ देखि ५)................................३<br>माध्यामिक (६ देखि १०/SLC)..................४<br>उच्च माध्यामिक (+२)...............................५<br>उच्च शिक्षा/बिश्व बिद्यालय.......................६	
२९	तपाईंको आमाले कति पढ्नु भएको छ ?	पढ्नु भएको छैन......................................१<br>अनौपचारिक शिक्षा...................................२<br>प्राथमिक (१ देखि ५).................................३<br>माध्यामिक (६ देखि १०/SLC)...................४<br>उच्च माध्यामिक (+२)................................५<br>उच्च शिक्षा/बिश्व बिद्यालय........................६	
३०	तपाईको आमा बुवा को मुख्य आम्दानीको श्रोत के हो?	कृषि.... ....१<br>ज्यालामजदुरी...२<br>सानो व्यापार/व्यवसाय...............................३<br>जागिर...४<br>घरभाँडा..५<br>अन्य (खुलाउनुहोस) ९६	
३१	तपाईको आमा बुवा को आफ्नो घर छ ?	छ..१<br>छैन..२ →	प्रश्न ३३

३२	तपाईको आमा बुवा को घरको छाना के को छ ?	खर/छवाली..१<br>टिन/जस्ता..२<br>टाईल/खपटा..३<br>सिमेन्ट..४<br>पलास्टीक/टेन्ट...५<br>अन्य (खुलाउनुहोस) ९६	
३३	तपाईको आमा बुवाको घरमा चर्पी छ ?	छ...१<br>छैन...२	प्रश्न ३५
३४	यदि छ भने कुन प्रकारको छ ?	अस्थाई...१<br>स्थाई...२<br>अन्य (खूलाउनुहोस) ९६	
३५	तपाईको आमा बुवाले कहाँको पानी प्रयोग गर्नुहुन्छ ?	पाइपको पानी (सार्वजनिक).............................१<br>पाइपको पानी (निजी)......................................२<br>टयूव वेल (सार्वजनिक)....................................३<br>टयूव वेल (निजी)..४<br>ईनार/कुवा (सार्वजनिक)..................................५<br>ईनार/कुवा (निजी)..६<br>अन्य (खुलाउनुहोस) ९६	
३६	के तपाईको माइतमा (आमा /बुवाले) गाईवस्तु पाल्नु भएको छ ?	छ...१<br>छैन...२	प्रश्न ३८ मा जाने
३७	के तपाईको माइतमा (आमा /बुवाले) निम्न उल्लेखीत जनावरहरु पाल्नु भएको छ ?<br><br>**(वहूउत्तर)**	भैसी.. ☐<br>गाई/गोरु.. ☐<br>घोंडा/गधा...................................... ☐<br>भेंडा/बाखा..................................... ☐<br>परेवा... ☐<br>कुखुरा/हाँस..................................... ☐<br>सूंगुर .... ☐<br>अन्य (खूलाउनुहोस) ९६	

३८	के तपाईको आमा बुवा को घरमा तलका सामानहरु छन् ?<br><br>**(वहूउत्तर)**	विजुली..१<br>मोटरबाईक.......................................२<br>रिक्सा...३<br>राँगागाडा वा वयलगाडा.........................४<br>साईकल..५<br>मोबाइल फोन.....................................६<br>टि.भी...७<br>रेडियो..८<br>पङ्खा...९<br>कम्प्यूटर..१०<br>फ्रिज..११	
**घ. व्यक्तिगत आनिबानी सम्बन्धि प्रश्नहरु**			
३९	तपाई कुनै कलव, स्थानिय हाट वजार मेला, भोजभतेर, नाचगान कार्यक्रमहरुमा रमाईलोको लागि जानु हुन्थ्यो ?	थियो..१<br>थिएन..२ →	प्रश्न ४१ मा जाने
४०	यदि जानु भएको भए प्राय कति पटक जानु हुन्थ्यो ?	नियमित...१<br>कहिलेकाहि...२<br>मैका मिलेमा ....३<br>एकपटक मात्र.....................................४ →	प्रश्न ४३ मा जाने
४१	तपाई कहिल्यै सिनेमा हेर्न जाने गर्नु भएको छ ?	छ...१<br>छैन..२	
४२	यदि जानु भएको भए प्राय कति पटक जानुभयो ?	नियमित...१<br>कहिलेकाहि...२<br>मैाका मिलेमा ....३<br>एकपटक मात्र.....................................४	
४३	तपाई कहिले काहीं मादक पदार्थ सेवन गर्नुहुन्छ ?	गर्दिन..१<br>एक पटक..२<br>मैका मिलेमा ....३<br>कहिलेकाहि...४<br>नियमित...५	

४४	तपाई चुरोट सेवन गर्नु हुन्छ ?	गर्दिन ....१<br>एक पटक...२<br>मैका मिलेमा ....३<br>कहिलेकाहि...४<br>नियमित..५	
**ङ. बिबाह तथा गर्भ सम्बन्धि प्रश्नहरु**			
४५	तपाईको बिबाहको प्रकार कस्तो थियो ?	प्रेम बिबाह/आफै..................................१<br>ववु आमाले दिएको................................२ →	प्रश्न ४८ मा जाने
४६	यदि प्रेम बिबाह भएको भए, तपाईले आफ्नो जीवन साथी कसरी भेटाउनु भयो ?	बिद्यालयको साथी.................................१<br>फेसबुक/सामाजिक संजालमा भेटेको ....२<br>साथीको माध्यमबाट भेटेको ....३<br>स्थानीय क्लबमा भेटेको ....४<br>.अन्य खुलाउनुहोस)..............................९६	
४७	यदि प्रेम बिबाह भएको भए, तपाई कसरी प्रभावित हुनु भयो ?	कसैबाट पनि होइन/आफै.......................१<br>टेलिभिजन/फिल्मबाट............................२<br>साथीअरुको दवाफबाट............................३<br>दुबैको सहमतिद्धारा.................................४<br>अन्य (खुलाउनुहोस)...............................९६	
४८	तंपाईको बिबाहको निर्णय कस्ले ग-यो ?	आफै...१ →<br>आमा/वुवाले..२<br>परिवारका अन्य सदस्यहरुले.........................३<br>अन्य (खुलाउनुहोस) ९६	प्रश्न ५० मा जाने
४९	तपांईको बिबाहको बारेमा तपांईलाइ सोधिएको थियो ?	थियो................. ....१<br>थिएन..२	
५०	तपाईको विवाह गर्दाको उमेर कति थियो ?	(पुरा उमेर बर्षमा) ☐☐	

५१	नेपालको कानुन अनुसार महिलाको लागि बिवाह गर्ने बैधानिक उमेर कति हो ?	थाहा छैन...१<br>१५ बर्ष...२<br>१६ बर्ष...३<br>१७ बर्ष...४<br>१८ बर्ष...५<br>१९ बर्ष...६<br>२० बष...७	
५२	तपाईको बिवाह गर्नुको कारण के थियो ?<br><br>**(वहूउत्तर)**	आर्थिक अवस्था कमजोर..........................१<br>ढिलो बिबाह गर्दा खर्चिलो हुने.. ....२<br>संस्कृति तथा रीतिरिवाज..........................३<br>अबैधानिक सम्बन्ध/बलत्कार/दुरब्यवहार......४<br>बिध्यालय छाडेको/फेल भएकोले.................५<br>अन्य (खुलाउनुहोस) ९६	
५३	उमेर नपुगि बिवाह गर्दा महिलामा हुनसक्ने असरहरुको बारेमा तपाईलाई थाहा छ ?	छ...१<br>छैन...२	→ **पश्न ५५**
५४	यदि थाहा छ भने ती असरहरु के के हुन् त ?<br><br>**(वहूउत्तर)**	पढाई छोड्नु पर्ने हुन्छ.............................१<br>छिटै बच्चा जन्मिनु.................................२<br>शरीरिक असर.......................................३<br>मानसिक असर......................................४<br>आत्मानिरभरतामा कमी............................५<br>अन्य (खुलाउनुहोस) ९६	
५५	तपाईको बच्चाहरु कति छन् ?	हाल सम्म बच्चा नपाएको/गर्भवति............१<br>एउटा..२<br>दुईवटा...३<br>तिनवटा...४	
५६	तपाई जम्मा कति पटक गर्भवती हुनुभयो ?	जम्मा गर्भवती संख्या ☐	
५७	तपाई पहिलो पटक गर्भवती हुँदां कति बर्षको हुनुहुन्थ्यो ?	बर्षमा ☐☐	

५८	तपाई जम्मा कति पटक गर्भवती हुंनु भयो ?	१<br>२<br>३	
५९	यो तंपाइको कुन पटकको गर्भ हो ?	१<br>२<br>३	
६०	यो तंपाइको योजना अनुसार वा इच्छाईएको गर्भ हो ?	योजना/चाहना अनुरुप रहेको गर्भ................१<br>चाहना बिपरित रहेको गर्भ...........................२	
६१	तपाईको बिचारमा युवतीहरु किन चाढै गर्भवती हुन्छन् ?<br>**(वहूउत्तर)**	गल्तीले ....१<br>होशियार नभएकोले ....२<br>केटा वा केटी साथी बनाउने ईच्छाले ....३<br>फाइदा हुने भएकोले ....४<br>आमा बन्ने ईच्छाले ....५<br>अन्य (खुलाउनुहोस)...................................९६	
६२	तपाई गर्भवती हुंन कसरी प्रभावित हुनु भयो ?<br>**(वहूउत्तर)**	साथीहरुको दवाफबाट.............................१<br>श्रीमान बाट.................. ....२<br>परिवारबाट.................. ....३<br>दुबैको सल्लाह अनुसार.............................४<br>सांकृतिक रितीरीवाज को कारणले................५<br>सामाजिक संजाल तथा सुचना....................६<br>अन्य (खुलाउनुहोस)................................९६	
६३	तपाई गर्भवती हुँदा वा बच्चा जन्माउँदा कुनै किसिमको स्वास्थ्य समस्या वा कठिनाइहरु भएको थियो ?	थियो...१<br>थिएन...२ →	प्रश्न ६५ मा जाने
६४	यदि थियो भने ती कठिनाइहरु के के थियो ?	गर्भपतन..१<br>महिना पुग्नु भन्दा अगाडि बच्चा जन्मेको ....२<br>कम तोल भएको बच्चा जन्मेको ....३<br>सुत्केरीकोबेला समस्या भएको ....४<br>पाठेघर खस्ने..५<br>यौन अंगको घाउ चोट पटक ....६<br>अपाङ्गता भएको बच्चा जन्मेको ....७<br>आमालाई अपाङ्गता बनाएको ....८<br>अन्य (खुलाउनुहोस).................................९६	

६५	गर्भवती हुँदा र बच्चा जम्माए पछि तपाईलाई बेखुशी वा नरमाइलो महसुस भएको थियो ?	थियो…………………………………………………१<br>थिएन…………………………………………………२ →	प्रश्न ६७ मा जाने
६६	यदि थियो भने ति के के थिए भन्न सक्नुहुन्छ ?<br>(२, ३ बुँदामा लेख्नुहोस)	………………………………………………………	
६७	चाढै गर्भवती भएको कारणले तपाईले बिद्यालय, परिवार, छिमेक र समाजमा कुनै समस्या वा कठिनाइहरुको सामना गर्नु परेको थियो ?	थियो…………………………………………………१<br>थिएन…………………………………………………२ →	प्रश्न ६९ मा जाने
६८	यदि थियो भने ति समस्या वा कठिनाइहरु के के थिए ?	बिद्यालयको पढाइ छोडनु परेको…………………१<br>बाआमा र परिवारमा अरुले अश्विकार गरेको…….२<br>लाजको महसुस गरेको ……………………………३<br>एक्लै भएको ………………………………………४<br>आर्थिक अभाव भएको ……………………………५<br>अन्य (खुलाउनुहोस)………………………………९६	
**च. यौन तथा HIV/AIDS रोगको ज्ञान सम्बन्धि प्रश्नहरु**			
६९	तपाईर्लाई यौन रोग तथा HIV/AIDS को बारेमा थाहा छ ?	छ……………………………………………………१<br>छैन……………………………………………………२ →	प्रश्न ७२ मा जाने
७०	एउटा ब्यक्तिलाई कसरी यौन तथा HIV/AIDS रोग लाग्न सक्छ ?	यौन सम्पर्कबाट………………………………………१<br>HIV/AIDS लागेको ब्यक्तिलाई छोएर………………२<br>HIV/AIDS लागेको ब्यक्तिले प्रयोग गरेको रुमाल तथा अन्य सामानहरु प्रयोग गरेर …………………………३<br>एउटै सुई तथा अन्य सामानहरुको पुन प्रयोग गरेर..४<br>बिरामीलाई रगत दिंदा………………………………५<br>गर्भधारनको बेला ……………………………………६<br>बच्चा जन्मदा आमाबाट बच्चामा सर्ने………………७<br>लामखुट्टे वा अन्य किराहरुको टोकाईबाट……………८<br>आमाको दुधबाट………………………………………९<br>अन्य (खुलाउने) ……………………………………९६	

७१	एउटा व्यक्तिले यौन रोग तथा HIV/AIDS बाट सुरक्षित रहन के गर्न सक्छ ?	बिबाह भन्दा पहिले यौन सम्पर्क नराख्ने ....१<br>धेरै जनासंग यौन सम्पर्क नराख्ने........................२<br>असुरक्षित यौन सम्पर्क नराख्ने...........................३<br>यौन सम्पर्क राख्ने काम पुर्णरुपमा बन्द गर्ने..........४<br>श्रीमानप्रति यौन स्रम्बन्धमा इमानदार रहने...........५<br>श्रीमानलाई पनि इमानदार रहन प्रोत्सहान गर्ने........६<br>दुषित रगतको प्रयोग नगर्ने..............................७<br>प्रत्येक पटकको यौन सम्पर्कमा कन्डमको प्रयोग गर्ने..८<br>एउटै सुई फेरि प्रयोग नगर्ने..............................९<br>अन्य (खुलाउने)..९६	
**छ. यौन, परिवार नियोजन तथा गर्भापतन सम्बन्धि प्रश्नहरु**			
७२	पहिलो पटक शारिरीक सम्पर्क हुँदा तपाईको उमेर कति थियो ?	पुरा गरेको बर्ष ☐	
७३	तपाईलाई परिवार नियोजनको साधनहरुको बारेमा थाहा छ ?	छ..१<br>छैन...२ →	प्रश्न ७८ मा जाने
७४	तपाईले पहिलो तथा त्यस पछिको शारिरीक सम्पर्कको बेला परिवार नियोजनको कुनै साधनको प्रयोग गर्नु भयो ?	पहिलो शारिरीक सम्पर्कको बेला प्रयोग नगरेको ......१<br>पहिलो शारिरीक सम्पर्कको बेला मात्र गरे..............२<br>पहिलो र त्यस पछिको शारिरीक सम्पर्कको बेला.......३<br>चाहेको गर्भ भन्दा अगडि सधै प्रयोग गरेको.. ....४<br>कहिंले प्रयोग नगरेको ....५<br>अन्य (खुलाउने) ९६	
७५	यदि गरेको छैन वा गर्नु हुन्न भने किन ?	मलाइ/मेरो श्रीमानलाई यसको प्रयोग मन पर्दैन...१<br>उपलब्ध नभएर ....२<br>यसले बाझोपना गर्छ ....३<br>स्वास्थ्य तथा महिनावरी चक्रमा असर... ....४<br>शरिरमा नकारात्मक असर ....५<br>किन्दा महँगो हुने भएकोले ....६<br>किन्ने पैसा नभएर..७<br>अर्को बच्चा बनाउने हाम्रो योजनाले गर्दा ....८<br>अन्य (खुलाउने) ९६	

७६	नियमित शारिरीक सम्पर्क वा असुरक्षित यौन सम्पर्क पछि तपाईले आकस्मिक परिवार नियोजनको कुनै साधन प्रयोग गर्नु भएको थियो?	गरेको थिंए...१<br>गरेको थिंईन २	
७७	कहिले प्रयोग गर्नु भएको थियो?	६ महिना अगाडि ....१<br>७ देखि १२ महिना अगाडि ....२<br>१३ महिना अगाडि ३	
७८	तपाईले कहिले गर्भपतन गर्नु भएको छ ?	गरेको छु..१<br>गरेको छैन..२→	प्रश्न ८१ मा जाने
७९	किन गर्भपतन गर्नु भएको ?	आफै गएको...१<br>अवैधानिक गर्भ भएकोले.................................२<br>चाढै गर्भ बसेको/मन नपरेकोले ....३<br>श्रीमानको दवावले गर्दा..................................४<br>परिवारको दवावले गर्दा..................................५<br>स्वास्थ्यको कारणले गर्दा.................................४<br>अन्य (खुलाउने)..९६	
८०	यदि गरेको हो भने कहाँ गर्नु भएको थियो ?	हस्पिटल/स्वास्थ्य संस्थामा..............................१<br>प्राइभेट क्लिनिक/फार्मेसीमा.............................२<br>परम्परागत उपाचर गर्ने ब्यक्तिहरु.....................३<br>अन्य (खुलाउने)..९६	
८१	गर्भपतनको असरको बारेमा थाहा छ?	छ..१<br>छैन...२	
८२	तपाईलाई उपयुक्त समय र हप्ता थाहा छ जुन अवधिभित्र सुरक्षित गर्भपतन गर्न सक्नु हुन्छ ?	१२ हप्ता अगाडि (मेडिकल गर्भपतन) ....१<br>१८ हप्ता अगाडि (बलत्कार)............................२<br>जुनसुकैबेला (स्वास्थ्यमा खतरा भएमा)................३<br>थाहा छैन..४<br>अन्य (खुलाउने)..९६	
**ज. महिला सशक्तिकरण सम्बन्धि प्रश्नहरु**			
८३	तपाई स्वास्थ्य चौकी, अस्पताल बजार र अन्य ठाउँहरुमा एक्लै जानु हुन्छ ? यसरी जाँदा कुनै रोकावाट हुन्छ ?	जान्छु...१<br>जान्न..२	
८४	बिरामी परेको बेला वा गर्भ जाँच्नको लागि स्वास्थ्य संस्थामा जान तपाई आफैले निर्णय गर्नसक्नु हुन्छ ?	सक्छु ....१ →<br>सक्दैन २	प्रश्न ८६ मा जाने

८५	यदि सक्दैन भने कसले निर्णय गर्छ ?	श्रीमान ....१<br>सासु ....२<br>ससुरा...३<br>अन्य (खुलाउने)..५	
८६	श्रीमान वा सासु ससुराको स्वीकृतीबिना दैनिक आवश्यक पर्ने सामानहरुको खरिदको लागि पैसा खर्च गर्नु सक्नु हुन्छ ?	सक्छु ....१<br>सक्दैन २	
**झ. हिंसा तथा शारिरिक/यौन शोषण सम्बन्धि प्रश्नहरु**			
८७	तपाईं आफ्नो परिवारसंग हुँदा सुरक्षित महसुस गर्नुहुन्छ ?	गर्छु ....१ →<br>गर्दिन २	प्रश्न ८९ मा जाने
८८	यदि सुरक्षित महसुस गर्नुहुन्न भने किन ?	शारिरीक शोषण को डर...........................१<br>मानसिक शोषण/यातना............................२<br>यौन दुब्यवहार..३	
८९	तपाईं कहिलै यौन शोषणको शिकार हुनुभएको छ ?	छ...१<br>छैन...२ →	प्रश्न ९४ मा जाने
९०	तपाई कति पटक यौन शोषणमा पर्नु भएको छ ?	नियमित. ....१<br>प्राय जसो ....२<br>कहिले काँही ....३<br>एकदम कममात्रमा ४	
९१	तपाईंलाई यौन शोषण गर्ने ब्यक्ति को थिए/हुन?<br>**(बहुत्तर)**	काम वा जागिर दिने/लगाईदिने...................१<br>श्रीमान ....२<br>परिवारको अन्य सदस्य ....३<br>सुरक्षाकर्मी ....४<br>साथी ....५<br>छिमेकी/समुदायको मान्छे. ....६<br>अन्य (खुलाउने) ७	
९२	पछिल्लो पटक तपाई यौन शोषणमा परेको घटनाबारे जिम्मेवार अभिभावकलाई भन्नुभएको थियो ?	भने ....१<br>भनिन २	

९३	यदि भन्नु भएन भने किन नभन्नु भएको हो?	भनेर केही पनि हुदैन ....१<br>कसैंले सुन्दैन ....२<br>डरले गर्दा ....३<br>लाजले गर्दा ....४<br>भन्ने कोही नभएर ....५<br>अन्य (खुलाउने) ....९६<br>थाहा नभएर ९९	
**ञ. अपाङगता सम्बन्धि प्रश्नहरु**			
९४	तपाईमा कुनै किसिमको अपाङ्गता छ ?	छ...१<br>छैन...२ →	रोक्ने
९५	यदि छ भने कस्तो किसिमको अपाङगता छ ?	दृष्टी सम्बन्धी...१<br>श्रवण सम्बन्धी..२<br>मानसिक..३<br>शारिरीक...४<br>वहू अपाङगता...५	
९६	तपाईमा अपाङ्गता कहिलेदेखि भएको थियो ?	जन्मैदेखि...८८८<br>जन्मेपछि (उमेर वर्षमा) .... [ ]	

धन्यबाद
